# Bone mineral density determinants in adolescents and young adults with congenital adrenal hyperplasia

**DOI:** 10.3389/fped.2024.1456679

**Published:** 2024-10-08

**Authors:** Ruta Navardauskaite, Aurika Vanckaviciene, Rasa Verkauskiene

**Affiliations:** ^1^ Department of Endocrinology, Medical Academy, Lithuanian University of Health Sciences, Kaunas, Lithuania; ^2^ Department of of Nursing, Medical Academy, Lithuanian University of Health Sciences, Kaunas, Lithuania; ^3^ Institute of Endocrinology, Medical Academy, Lithuanian University of Health Sciences, Kaunas, Lithuania

**Keywords:** congenital adrenal hyperplasia, CAH, bone health, bone mineral density, transition age

## Abstract

**Background:**

The effects of long-term glucocorticoid (GC) treatment on bone mineral density (BMD) in patients with congenital adrenal hyperplasia (CAH) remain controversial.

**Objectives:**

This cross-sectional study aimed to evaluate BMD in relation to genotype, growth, vitamin D status, cumulative GC doses, and other relevant factors in youths with CAH.

**Methods:**

Thirty-two patients with classical CAH (13 males; mean age 26.0 ± 7.1 years) were compared with 32 healthy controls matched by age and sex. BMD was measured using dual-energy x-ray absorptiometry, and statistical analyses, including the Mann–Whitney *U*-test and Spearman's correlation coefficient, were performed to evaluate differences and associations.

**Results:**

Median whole-body and lumbar BMD *Z*-scores were similar between CAH patients and controls (*p* = 0.27 and 0.15, respectively). Low bone density was observed in 12.5% of CAH patients and 18.75% of controls (*p* = 0.5), and osteoporosis was confirmed in 12.5% of CAH patients and 0% of controls (*p* = 0.04). BMD did not correlate with cumulative GC doses, estradiol, renin, phosphate, sodium levels, or anthropometric parameters in CAH patients. There was no significant difference in BMD between severe and non-severe genotypes of CAH. However, a positive correlation was found between the whole-body BMD *Z*-score and growth velocity during infancy (*r* = 0.776, *p* = 0.021) in CAH patients. Vitamin D deficiency was noted in 56.25% of CAH patients, although vitamin D levels did not correlate with BMD or genotype. No history of bone fractures was reported among study participants.

**Conclusions:**

CAH patients are at risk of developing osteoporosis, but in this study, BMD *Z*-scores were not associated with cumulative GC doses. The study did not identify an association between genotype and BMD. Poor growth during infancy was linked to decreased BMD in adulthood.

## 1 Introduction

Osteopenia and osteoporosis are systemic skeletal diseases characterized by low bone mass, microarchitectural deterioration of bone tissue leading to increased bone fragility, and a consequent increase in fracture risk (1). Low bone density is usually the result of accelerated bone turnover due to estrogen deficiency, whereas in aging women and men, vitamin D insufficiency and secondary hyperparathyroidism may further contribute to bone loss and are not frequent in adolescents and young adults. However, the etiology, diagnosis, and treatment of osteoporosis in adolescents and adults under 50 years of age remain poorly defined (2).

Along with heredity factors that affect genes and their polymorphisms and account for 50%–80% of the variation in bone mass and structure among individuals (3), endocrine, nutritional, and other risk factors, such as low levels of physical activity, delayed puberty or secondary amenorrhea, systemic inflammation, or long-term corticosteroid treatment, precipitate bone loss at a younger age (3).

Congenital adrenal hyperplasia (CAH) is an autosomal recessive disorder that results from impaired steroidogenesis in the adrenal cortex (4, 5). In 95% of cases, CAH is caused by mutation of the *CYP21A2* gene, which encodes the enzyme 21-hydroxylase (21OH) (6, 7). Owing to a deficiency in 21OH (21OHD), the synthesis of cortisol and, in severe forms, aldosterone is impaired. The treatment of CAH requires the replacement of glucocorticoid (GC) and mineralocorticoid (MC) therapy, balancing between adrenal insufficiency and androgen excess (8); therefore, in most cases, the therapeutic doses of GC are supraphysiological. Low BMD has been detected in 40%–60% of CAH cases (9–11); however, data on its association with GC type and dosage and other presumed risk factors are conflicting (12, 13).

Vitamin D deficiency is known to be associated with low bone mineral density (BMD) in the general population (14) and is common (50%–80%) in CAH patients (10, 15); however, only a few studies have reported the association of vitamin D levels with BMD in CAH patients (16). In addition, it has previously been shown that early growth patterns are predictive of BMD status in later life (17, 18). In our previous study, we demonstrated that patients with classical CAH forms have different GC dose requirements during infancy dependent on the genotype and residual 21OH secretion and hypothesized that it may affect their early postnatal growth pattern (19). Therefore, in the present study, we aimed to analyze BMD in relation to genotype, growth, vitamin D status, cumulative GC doses, and other relevant factors in adolescents and young adult patients with classical forms of CAH in comparison with healthy controls.

## 2 Patients and methods

### 2.1 Subjects

All patients older than 14 years of age in the Lithuanian database of CAH patients were invited to participate in the study conducted by the Lithuanian University of Health Sciences (Kaunas, Lithuania) from 2018 to 2022. All patients included in the study were born before the establishment of the Newborn Screening Program for CAH in Lithuania (20). We included patients with classical CAH diagnosed through genetic testing and excluded those with other endocrine disorders that might affect bone density. Thirty-two adolescents and young adults (14–37 years of age) with CAH were recruited and compared with 32 healthy control subjects rigorously matched for age, sex, pubertal stage, and ethnicity. Eight patients each in the CAH and control groups were adolescents (14–18 years old). Puberty was evaluated according to the Tanner stage (21).

### 2.2 Physical examination

Anthropometric measures of height (cm) and weight (kg) were obtained from all participants. Height and sitting height were measured using a Harpenden stadiometer. The height standard deviation score (Ht-SDS) was calculated according to age and gender using Lithuanian National Children Growth Evaluation Chart references for all participants under 18 years of age (22). The sitting height and height (SitHt/Ht) ratio was calculated and adjusted for age and pubertal stage. The body mass index (BMI) SDS was calculated using Lithuanian National Children Growth Evaluation Chart references (22). A BMI between 25 and 29.9 kg/m^2^ (>+1 SD and <+2 SD) was classified as overweight, and a BMI of 30 kg/m^2^ or more (>+2 SD) was classified as obesity. Growth velocity (cm/year) was evaluated in patients with CAH across three distinct periods from birth to final adult height: (1) Infancy—from birth to 1 year of age; (2) Childhood—from 1 year of age until the appearance of the first signs of puberty; (3) Puberty—from the onset of puberty until final adult height was reached. Medical records of growth evaluation were available for 20 CAH patients (6 males and 15 salt wasters).

### 2.3 Biochemical and molecular diagnosis of CAH

The diagnosis of 21OHD was confirmed through mutation analysis of the *CYP21A2* gene. All patients were genotyped for confirmation of the diagnosis. *CYP21A2* gene and *CYP21A1P* pseudogene copy number analysis was performed using quantitative multiplex ligation-dependent probe amplification (MLPA) with SALSA® MLPA® probemix P050-C1 CAH [MRC-Holland, Amsterdam, The Netherlands; reference sample—SD039-S02 Reference DNA (MRC-Holland)]. The detection of sequence changes in the *CYP21A2* gene was performed using Sanger sequencing after selective long-range PCR with primers specific for *CYP21A2* and/or *CYP21A1P* (23).

The definition of CAH forms was based on the *CYP21A2* genotype, initial and follow-up plasma renin concentrations, and electrolyte status at diagnosis: severe *CYP21A2* mutations on both alleles or complete gene deletions/conversions, significantly elevated renin concentrations, or apparent salt loss at the time of CAH diagnosis were indicative of the salt-wasting (SW) form of CAH. Patients with severe *CYP21A2* mutations on one allele and mild mutations on another allele causing mild 21OHD with normal or only slightly elevated renin concentrations and normal electrolyte levels were diagnosed with the simple virilizing (SV) form of CAH. The stratification of common *CYP21A2* pathogenic variants by residual enzyme activity was based on Krone et al. and Concolino and Costella (24, 25) and is detailed in Table 1.

**Table 1:** Grouping of common *CYP21A2* pathogenic variants by residual enzyme activity**.**

Enzyme activity (%)	Phenotype	*CYP21A2* pathogenic variant	Grouping of mutations according to 21OH activity
0	Severe (classic)	Whole-gene deletion Large- gene conversion p.Gly111ValfsTer21 p.[Ile237Asn;Val238Glu;Met240Lys] p.Leu308PhefsTer6 p.Gln319Ter p.Arg357Trp	Null
<1		c.293-13A>G c.293C>G	A
2–11		p.Ile173Asn	B
∼20–50	Mild (non-classic)	p.Pro31Leu p.Val282Leu p.Pro454Ser	C

21OH, 21-hydroxylase.

Genotypes were classified according to residual 21OH activity (Null, A, B, and C). For further analysis, CAH patients were divided into two subgroups according to genotype: the first subgroup (*n* = 17) included patients with mutations that caused 0% or close to 0% 21OH activity (null mutations in both alleles or null mutations in one allele and an A type mutation in another allele), and the second subgroup (*n* = 15) included other combinations of mutations associated with ≥1% of the residual 21OH activity.

### 2.4 Therapy

GC doses were expressed as the cumulative dose per body surface (mg/m^2^/day). The median dose of GC was calculated as the actual cumulative corticosteroid dose during all treatment periods. All the GC doses used were converted into hydrocortisone (HC) dose equivalents for the purpose of normalization using anti-inflammatory equivalents [20 mg of hydrocortisone = 5 mg of prednisolone (PD) = 0.75 mg of dexamethasone (DEX)] (26). Five patients (15.6%) were treated with PD and six (18.8%) with DEX; the rest were treated with HC.

Treatment efficacy was assessed by serum 17-hydroxyprogesterone (17OHP, 12–32 nmol/L) and testosterone (T; normal T levels were evaluated according to the chronological age and sex in at least two of four annual measurements) and adrenocorticotropic hormone (ACTH; normal range 1.63–14.15 pmol/L) measurements. MC replacement was monitored by blood pressure and renin concentration (normal range 1.6–14.7 ng/L) and was maintained within the upper normal limit.

The vitamin D level was not evaluated routinely for the patients before this study and one-third of the patients periodically used supplements containing vitamin D, often at a dose of 600–1,000 IU/day. Information about the use of vitamin D supplements was not collected from the controls.

One female with SW CAH had nephrocalcinosis. All the studied subjects who had normal thyroid function did not use thyroid-function-affected medication. No patient used any additional medications that affected bone mineralization or structure (e.g., aromatase inhibitors to increase adult height) (2). There was no history of bone fractures in any of the study groups.

### 2.5 Laboratory investigations

Fasting blood samples were taken in all study subjects before GC and fludrocortisone administrations, between 8:00 and 9:00 am, from the antecubital vein catheter for measurements of 17OHP, T, ACTH, renin, 25OH-vitamin D, parathormone (PTH), alkaline phosphatase (ALP), calcium (Ca), phosphate (P), and sodium (Na).

Vitamin D deficiency was defined as a 25OH-vitamin D concentration below 50 nmol/L (20 ng/ml) and vitamin D insufficiency was defined as 25OH-vitamin D levels between 50 and 75 nmol/L (21–29 ng/ml) (27). The normal reference range of PTH levels was defined as 1.26–6.97 pmol/L. The normal reference range for ALP varies depending on age and sex; the normal range was defined as 30–120 U/L for participants (young adults) in our study.

Hyponatremia was defined as an Na concentration below 136 mmol/L.

### 2.6 Bone mineral density measurement

Whole-body dual-energy x-ray absorptiometry (DXA; Hologic, Marlborough, MA, USA) was used to measure total body BMD, and lumbar vertebra (L1–L4) BMD. Bone mineral density values were used to calculate BMD *Z*-scores according to chronological age. According to the International Society for Clinical Densitometry (ISCD) 2007 Pediatric Official Positions and current literature, in children, low BMD was defined as a *Z*-score of less than −2.0 SD at the lumbar spine and/or whole body adjusted for age, gender, and body size, as appropriate (28). For adults, low BMD was defined as a *T*-score of less than −1.0 and at least −2.5 SD, and osteoporosis was defined as a *T*-score less than of −2.5 SD at the femoral neck and lumbar spine (L1–L4) according to World Health Organization criteria (29).

### 2.7 Biochemical and hormonal assays

Concentrations of testosterone (nmol/L; Biosource, Belgium), 17-hydroxyprogesterone (nmol/L; DIAsource, Belgium), adrenocorticotropic hormone (pmol/L; DIAsource, Belgium), dehydroepiandrosterone sulfate (DHEAS; μmol/L, Stratec biomedical systems, Germany), renin (ng/L; DIAsource, Belgium), parathormone (pmol/L; DIAsource, Belgium), alkaline phosphatase (U/L; Beckman Coulter Prague, Czech Republic), calcium (mmol/L; Beckman Coulter, Prague, Czech Republic), phosphate (mmol/L; Beckman Coulter, Prague, Czech Republic), sodium (mmol/L; Beckman Coulter, Prague, Czech Republic), and 25OH-vitamin D (nmol/L; Tosoh Corporation, Japan) were measured using an immunoradiometric assay.

### 2.8 Bioethics

The study was approved by the Kaunas Regional Ethics Committee of Biomedical Research (No BE-2–29, approved 23 April 2018). All procedures were carried out with the adequate understanding and written consent of the participants. For participants below 18 years of age, written consent from their parents or caregivers was also obtained. The investigation was carried out in accordance with the Declaration of Helsinki.

### 2.9 Statistics

Statistical analyses were performed using SPSS version 29.0 (IBM Corp., Armonk, NY, USA). Given the non-parametric nature of our data, several statistical tests were employed to evaluate the relationships between variables.

The Mann–Whitney *U*-test was selected for comparing two independent groups (e.g., CAH patients vs. controls and SW CAH vs. SV CAH) due to its robustness when dealing with non-normally distributed data. Unlike parametric tests such as the *t*-test, the Mann–Whitney *U*-test does not assume normality in the data, making it appropriate for the small sample sizes and skewed distributions observed in our study. This test ranks all the values from both groups together and then assesses whether the ranks differ significantly between the groups, providing a *p*-value that indicates whether there is a statistically significant difference in the central tendencies of the two groups.

Spearman's correlation coefficient was used to assess the strength and direction of association between two continuous or ordinal variables. This non-parametric measure is particularly suited for our study as it does not assume a linear relationship between variables and is less sensitive to outliers than Pearson's correlation coefficient. Spearman's correlation ranks the values of the variables and then calculates the correlation based on these ranks, providing a correlation coefficient (*r*) that ranges from −1 to 1. A positive value indicates a direct relationship, whereas a negative value indicates an inverse relationship. The significance of the correlation is determined by the associated *p*-value, with *p* < 0.05 considered statistically significant in our analyses.

Continuous variables were presented as medians with interquartile ranges (IQR) or means with standard deviations (SD) depending on their distribution. Before selecting the appropriate statistical test, the normality of the data distribution was evaluated using the Shapiro–Wilk test. Given that many variables did not follow a normal distribution, non-parametric tests were generally favored.

## 3 Results

**Figure 1:**
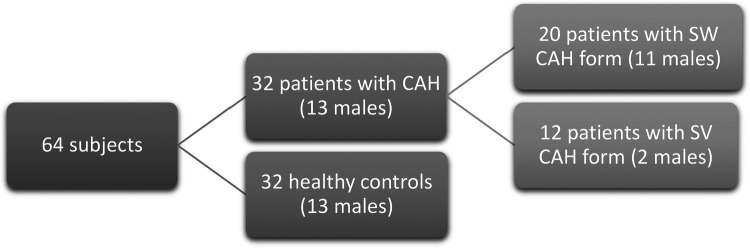
Descriptive scheme of the study subjects. The study subjects were 32 CAH patients and 32 healthy controls. CAH, congenital adrenal hyperplasia; SW CAH, salt-wasting CAH form; SV CAH, simple virilizing CAH form.

Twenty (60%) patients had the SW form of CAH and 12 patients (40%) had the SV form of CAH (Figure 1). In each study group, one patient (12.5%) was in Tanner stage 3, three (37.5%) were in Tanner stage 4, and four (50%) were in Tanner stage 5.

### 3.1 Bone mineral density and other clinical characteristics

CAH patient and healthy control characteristics are presented in Table 2. The median of whole-body and lumbar BMD *Z*-scores did not differ between the CAH and control groups (*p* = 0.27 and 0.15, respectively), but osteoporosis (whole-body BMD *Z*-score < −2 SD) was detected only in CAH patients (12.5% vs. 0%, *p* = 0.04, respectively). The frequency of low BMD, defined as a BMD *Z*-score between −2.5 and −1 SD, was detected in 18.75% of controls and 12.5% of CAH patients (*p* = 0.5). CAH patients were significantly shorter than controls (*p* < 0.001), had a higher BMI-SDS (*p* < 0.001), and a higher total fat mass (TFM) *Z*-score (*p* = 0.003).

**Table 2:** Clinical characteristics of the CAH patients and matched controls.

Variables	SW males (*n* = 11)	SV males (*n* = 2)	SW females (*n* = 9)	SV females (*n* = 10)	SW (*n* = 20)	SV (*n* = 12)	All CAH (*n* = 32)	Controls (*n* = 32)
Age (years)	26.6 (16.9 to 29.2)	30.2 (28.9 to 30.45)	24.3 (20.8 to 28.4)	30.9 (20.9 to 36.5)	25.0 (17.1 to 29.2)***	30.9 (23.7 to 36.0)***	26.9 (17.9 to 31.6)	28.6 (17.1 to 31.3)
Whole-body BMD (*Z*-score)^a^	−0.9 (−1.45 to −0.17)*	−3.4 (−3.4 to −22.7)*	−0.95 (−1.2 to −0.45)	−0.4 (−2.2 to 0.2)	−0.95 (−1.3 to −0.32)	−0.65 (−2.35 to 0.13)	−0.9 (−1.45 to −0.15)	−0.6 (−1.3 to −0.15)
Lumbar BMD (*Z*-score)^a^	−0.5 (−0.8 to −0.1)	−1.75 (−2.9 to −1.75)	−0.3 (−0.8 to 0.5)	−0.35 (−1.07 to 0.3)	−0.4 (−0.8 to 0.05)	−0.55 (−1.5 to 0.1)	−0.45 (−0.9 to 0.03)	−0.4 (−0.85 to 0.1)
Median cumulative equivalent HC dose (mg/m^2^/day)	16.6 (14.2 to 17.7)	13.5 (13.3 to 13.5)	16.8 (13.6 to 20.7)	14.4 (12.3 to 16.1)	16.6 (14.1 to 18.5)***	13.9 (12.6 to 15.2)***	15.4 (13.2 to 17.7)	
Median cumulative MC dose (μg/day)^a^	100 (87.5 to 125)		100 (50 to 100)		100 (50 to 125)			
BMI-SDS^b^	0.31 (−0.4 to 1.87)	2.28 (1.24 to 2.5)	1.84 (1.14 to 3.01)**	1.8 (1.7 to 2.63)**	0.93 (−0.03 to 2.17)	1.98 (1.57 to 2.82)	1.63 (0.3 to 2.4) ****	0.41 (−0.68 to 1.19)****
Ht-SDS^c^	−1.47 (−2.13 to −0.75)*	−2.11 (−2.4 to −2.11)*	−0.69 (−1.28 to 0.1)**	−1.13 (−3.29 to −1.13)**	−1.14 (−1.18 to −0.58)	−1.76 (−2.58 to −1.15)	−1.13 (−2.3 to −0.79)****	0.19 (−0.84 to 1.03)****
Vitamin D (nmol/L)	54.5 (31.4 to 80.5)	36.8 (36.8 to 38.6)	45.9 (35.5 to 76.7)	39.3 (29.0 to 59.7)	48.9 (0.41 to 0.55)	36.8 (29.1 to 56.8)	45.2 (30.6 to 70.3)	57.8 (40.9 to 69.4)
Calcium (mmol/L)	2.35 (2.19 to 2.45)	2.39 (2.39 to 2.39)	2.36 (2.23 to 2.4)	2.22 (2.17 to 2.28)	2.35 (2.2 to 2.43)	2.25 (2.2 to 2.32)	2.28 (2.2 to 2.42)	2.4 (2.32 to 2.46)
Phosphate (mmol/L)	1.1 (0.94 to 1.3)	1.25 (1.25 to 1.25)	1.12 (0.81 to 1.3)	1.12 (1.06 to 1.23)	1.1 (0.92 to 1.3)	1.17 (1.08 to 1.23)	1.15 (0.97 to 1.28)	1.04 (0.93 to 1.16)
Sodium (mmol/L)	138 (136 to 141)	136 (136 to 136)	139 (135.3 to 139)	137 (133.2 to 138.8)	138 (136 to 139)	136 (133.5 to 138.5)	138 (135.2 to 139)	139 (137 to 140)
T (nmol/L)^b^	19.1 (8.3 to 23.9)	9.7 (7.5 to 10.2)	3.78 (1.54 to 5.46)	1.74 (0.52 to 4.7)	14.4 (5.1 to 22.5)***	1.87 (0.84 to 6.48)***	7.08 (2.7 to 19.3)	1.99 (1.39 to 14.9)
17OHP (nmol/L)^b^	124.2 (20.3 to 420)	61.5 (4.1 to 70.6)	434 (258 to 472)	229 (20.7 to 410)	161 (35.2 to 520)	119 (20.6 to 329)	156 (20 to 410)	
Renin (ng/L)^b^	13.7 (4.0 to 29.4)	9.2 (4.5 to 10.2)	28.3 (7.5 to 57.8)	14.9 (9.1 to 21.8)	13.7 (5.6 to 31.4)	14.6 (9.0 to 19.8)	14.6 96.8 to 29.1)	
SHBG (nmol/L)	32.8 (25.5 to 40.6)	39.9 (27 to 39.9)	49.0 (32.5 to 74.5)	66.5 (38.7 to 179.0)	34.5 (27.3 to 47.0)	48.4 (34.5 to 127.1)	40.5 (29.3 to 49.0)	
TFM (kg)^d^	16.2 (13.9 to 18.9)*	32.4 (19.7 to 35.7)*	29.4 (18.5 to 32.4)**	25.2 (24.6 to 37.4)**	17.5 (14.6 to 23.5)***	25.2 (23.5 to 37.4)***	22.5 (16.2 to 27.1)	19.3 (16.8 to 24.7)
TFM (*Z*-score)^c^	0.4 (0.2 to 0.5)	1.25 (0.1 to 1.3)	0.8 (−0.2 to 0.82)**	0.3 (0.2 to 1.1)**	0.45 (0.12 to 0.8)	0.3 (0.15 to 1.2)	0.4 (0.2 to 0.8)****	0.05 (−0.5 to 0.27)****
VAT (grams)^c^	274 (252 to 359)*	750 (360 to 800)*	434 (197 to 450)**	543 (247 to 566)**	283 (249 to 387)	543 (266 to 662)	348 (252 to 501)	293 (201 to 358)
SAT (kg)^c^	16.0 (13.7 to 18.5)*	31.7 (19.3 to 32.6)*	28.9 (18.3 to 30.5)**	25.0 (24.3 to 36.6)**	17.3 (14.4 to 23.1)***	25.0 (23.3 to 36.8)***	22.3 (16.0 to 26.6)	18.9 (16.6 to 18.9)
Lean body mass (kg)^c^	43.3 (40.0 to 52.6)	56.5 (54.1 to 58.5)	41.1 (37.1 to 43.2)	41.0 (39.6 to 43.3)	43.3 (39.7 to 52.8)	43.1 (39.7 to 51.5)	43.2 (39.7 to 52.6)	44.3 (36.6 to 55.6)

CAH, congenital adrenal hyperplasia; SW, salt-wasting; SV, simple virilizing; M, males; F, females; HC, hydrocortisone; MC, mineralocorticoid; BMI-SDS, body mass index by standard deviation score; Ht-SDS, height by standard deviation score; BMD, bone mineral density; T, testosterone; 17OHP, 17-hydroxyprogesterone; TFM, total fat mass in kilograms; kg, kilograms; VAT, visceral abdominal tissue mass; SAT, subcutaneous adipose tissue; SHBG, sex hormone binding globulin.

Values are represented as median (25th–75th percentile).

* *p* < 0.05 between the SW and SV CAH males subgroups.

** *p* < 0.05 between the SW and SV CAH females subgroups.

*** *p* < 0.05 between the SW and SV CAH subgroups.

**** *p* < 0.05 observed between the CAH and controls subgroups.

^a^ Adjusted for age, pubertal stage, body surface area.

^b^ Adjusted for age, pubertal stage.

^c^ Adjusted for age, gender.

^d^ Adjusted for age, Ht-SDS.

The median of vitamin D level was 45.2 (30.6–70.3) nmol/L in CAH patients and 57.8 (40.9–69.4) nmol/L in the control group (*p* = 0.35). Eighteen (56.3%) patients and 13 (40%) controls had vitamin D deficiency (*p* = 0.21). BMD Z-scores did not significantly correlate with vitamin D levels (*p* > 0.05). The median levels of PTH (2.08–5.78 pmol/L) and ALP (48–113) were within the normal range across all subject groups. Significant differences and correlations between variables were not detected between the groups. There was no history of bone fractures in CAH patients and the controls.

### 3.2 The relationship between bone mineral density and other clinical and genetic factors

We identified significant associations between whole-body BMD and the BMD of separate body segments with T levels in the whole study cohort (Table 3). We did not observe any significant correlations between BMD and other clinical or biochemical parameters [17OHP, T, estradiol, renin, DHEAS, sex hormone binding globulin (SHBG), vitamin D, calcium, phosphate and sodium levels, age, height and achieved final height, SitHt/Ht ratio, BMI-SDS, TFM, visceral abdominal tissue (VAT), subcutaneous adipose tissue (SAT), or lean body mass].

**Table 3:** Relationship between BMD and testosterone levels in the whole study group.

	Spearman's rho	*p*-value
Total BMD (g/cm^2^)	0.295	0.042
Subtotal BMD (*Z*-score)^a^	0.422	0.003
Ribs BMD (*Z*-score)	0.486	0.001
Right arm BMD (g/cm^2^)	0.519	0.001
Left arm BMD (g/cm^2^)	0.455	0.001

^a^ Subtotal BMD: total body BMD excludes head.

When analyzing the whole CAH patient group separately, we did not find significant associations between BMD and others variables (17OHP, T, estradiol, renin, DHEAS, SHBG, vitamin D, calcium, phosphate and sodium levels, age, height and achieved final height, SitHt/Ht ratio, BMI-SDS, TFM, VAT, SAT, or lean body mass). The final height of patients with SW and SV CAH was lower than controls. Patients did not achieve their target height.

Analyzing CAH patient subgroups separately, total BMD (g/cm^2^) was significantly related to SAT mass (*r* = 0.773, *p* = 0.005) in males with the SW form, although the median of SAT mass was higher in the SV group than in the SW group [31.7 (19.3–32.6) kg vs. 16.0 (13.7–18.5) kg (*p* = 0.013), respectively].

In males with CAH, whole-body BMD *Z*-score and total BMD in g/cm^2^ were directly associated with 17OHP concentration [*r* = 0.74 (*p* = 0.006) and *r* = 0.59 (*p* = 0.026), respectively]; total BMD in g/cm^2^ was also significantly related to DHEAS levels (*r* = 0.695, *p* = 0.012), and the lumbar BMD *Z*-score was inversely related to SHBG levels (*r* = −0.669, *p* = 0.017). In females with the SW form of CAH, the lumbar BMD *Z*-score was inversely related to calcium levels (*r* = −0.9, *p* = 0.037).

Patients with the SW form of CAH were treated with significantly higher median cumulative equivalent HC doses than patients with the SV form of CAH [16.6 (14.1–18.5) and 13.9 (12.6–15.2) mg/m^2^/day, respectively, *p* < 0.05] (Table 2), but cumulative equivalent HC doses were not related to BMD.

The correlation between genotype and expected phenotype and the positive predictive value of this cohort have been described previously (19). No significant differences were found in the BMD *Z*-scores or BMD (g/cm^2^), but a significantly higher vitamin D concentration was identified in the Null/Null and Null/A group compared with the other group with the mildest genotype [64.6 (25.2–107) vs. 38.0 (21.7–70.3), respectively, *p* = 0.003]. We did not find any differences in the other analyzed clinical and biochemical parameters when comparing CAH patients carrying Null/Null and Null/A mutations with other genotypes.

**Figure 2:**
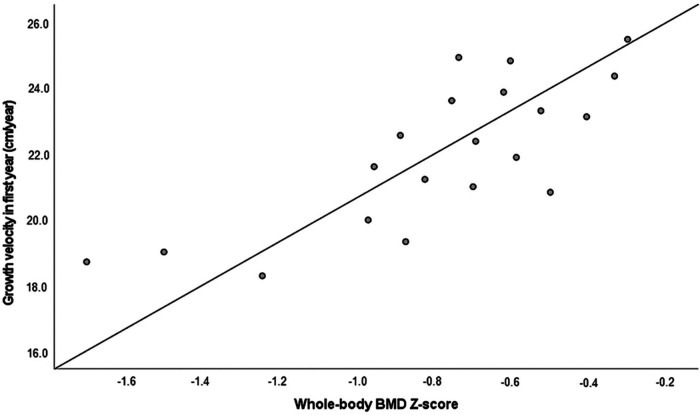
The whole-body BMD *Z*-score in association with growth velocity during the first year of life (*r* = 0.776, *p* = 0.021, *n* = 20). BMD, bone mineral density.

We analyzed the association of BMD and linear growth in different periods of postnatal growth in CAH patients. A positive strong correlation was found between the whole-body BMD *Z*-score and growth velocity (cm/years) in infancy (from birth until the first year of life) (*r* = 0.776, *p* = 0.021) (Figure 2). No significant correlations were identified between BMD and growth velocity during childhood (from 1 year until puberty onset) or puberty (from the first signs of puberty until the achieved final height).

The sodium level for CAH patients was within target limits throughout the whole treatment period. Chronic hyponatremia was not observed in any patient. The median sodium concentration during the whole treatment period was 137 (136–138) mmol/L and did not differ between the CAH subgroups (*p* = 0.53). Sodium concentrations at the cross-sectional study visit, sodium concentrations at diagnosis, and sodium concentrations throughout the whole treatment period were not associated with BMD *Z*-scores and GC and MC doses.

## 4 Discussion

We identified osteoporosis in 12.5% of CAH patients, which was significantly more frequent than in healthy controls. In our study, the prevalence of osteoporosis was one of the lowest compared with other studies on CAH patients, in which the prevalence varied between 13% and 52% (29–34). Of note, only a few studies evaluated BMD in young CAH patients with the SW form of the disease (30–32, 34). One of the largest studies in the USA reported normal BMD in the majority of children with CAH (10).

Some studies have indicated that GC therapy is a possible contributor to decreased BMD and osteoporosis in CAH patients (4, 15, 33). However, in our study, BMD was not associated with cumulative doses of GC. A systematic review and meta-analysis of BMD in adults with CAH presented analyses of studies and the majority of them did not observe correlations between GC doses and BMD, regardless of the high variation in HC equivalent doses (9.66–22 mg/m^2^/day) (35).

Similarly, in a study by Finkielstain et al., adult patients with low BMD (a *Z*-score ≤ 2) did not differ from patients with normal BMD in terms of age, sex, CAH phenotype, or GC doses; only 17OHP was lower (*p* = 0.007) in patients with low BMD (10).

Based on the relationship of gonadal and adrenal androgens with osteoblasts as stimulators of proliferation and differentiation in both males and females (36), we aimed to analyze this association in our study. We determined the correlation between T and BMD in all subjects (patients and controls) and a strong positive correlation between DHEAS and BMD in the male CAH group (37). Some studies found an association between the failure to have a physiological increase in DHEAS levels during adrenarche and low BMD (38). This relationship is based on the result of a GC overdose, which affected growth and osteoblastic function in these patients and might be the cause of low BMD.

In addition, recent research has focused on the role of adrenal androgens, particularly DHEAS, in bone health. Our study found a significant association between DHEAS levels and BMD in male CAH patients, consistent with recent findings that suggest adrenal androgens may play a role in bone density through their effects on osteoblast function. However, the complex interplay between GC therapy, androgen levels, and bone health suggests the need for further research to better understand these relationships and develop targeted interventions to preserve bone health in CAH patients (39).

In our study, we analyzed the relationship between BMD and growth in various periods of life. We determined a strong positive association between whole-body BMD and growth velocity during infancy. This finding confirmed the conclusion of previous studies that poor growth during early life is linked to a number of adverse health outcomes in adulthood, including decreased adult bone mass (17, 40).

In recent years, the impact of GC therapy on BMD in patients with CAH has been extensively studied, yet the results remain inconsistent. Although some studies suggest a negative impact of higher cumulative GC doses on BMD, others have not found a significant correlation. A recent meta-analysis by Falhammar et al. in (41) found that the relationship between GC doses and BMD in CAH patients is variable, with individual responses likely influenced by factors such as genotype, adherence to therapy, and early-life growth patterns (39).

Emerging evidence indicates that early-life growth is a critical determinant of BMD in adulthood. Several studies have shown that suboptimal growth during infancy, often due to adrenal crises or inadequate early GC therapy, is associated with lower BMD in later life. This association underscores the importance of optimizing early growth to prevent long-term skeletal complications. Our study aligns with these findings, as we observed a strong positive correlation between growth velocity during the first year of life and whole-body BMD in adulthood, highlighting the need for personalized GC dosing strategies during infancy to mitigate future risks (42).

Sweden's study analyzed a high number of patients with 21OHD and presented data showing a high incidence of fractures. Significantly higher frequencies of fractures were determined in patients born before the introduction of neonatal screening but not in those born afterward, which presumes the association between late diagnosed CAH and a higher GC dose in infancy due to developed adrenal crisis. It could support our determined correlation between BMD and growth velocity during the first year of life. However, in the same study, the highest prevalence of fractures was observed in the SV phenotype and I172N genotype, whereas the I2 splice (determinant SW form) genotype did not show an increased prevalence (42), the delayed diagnosis of the SV form later in childhood and lower BMD in adulthood were not related to the reduced growth velocity during the early years.

In conclusion, our study provides valuable insights into the factors influencing bone mineral density in adolescents and young adults with congenital adrenal hyperplasia. Although no significant correlation was found between cumulative glucocorticoid doses and BMD, our findings highlight the importance of early-life growth patterns in determining adult bone health. The positive association between growth velocity during infancy and BMD underscores the need for optimized CAH management from early life to prevent long-term skeletal complications.

Furthermore, the observed relationship between DHEAS levels and BMD in male patients suggests that adrenal androgens may play a role in bone health, warranting further investigation. These findings have important clinical implications, suggesting that more personalized approaches to GC therapy, considering both genetic and early growth factors, may be beneficial in preserving bone health in CAH patients.

Future research should focus on identifying specific factors that contribute to the variability in bone health outcomes among CAH patients and explore potential strategies to mitigate the adverse effects of chronic GC therapy on bone. This will be crucial in improving the overall quality of life for individuals with CAH as they transition into adulthood.

## Data Availability

The original contributions presented in the study are included in the article/Supplementary Material; further inquiries can be directed to the corresponding author.
